# In silico MS/MS spectra for identifying unknowns: a critical examination using CFM-ID algorithms and ENTACT mixture samples

**DOI:** 10.1007/s00216-019-02351-7

**Published:** 2020-01-22

**Authors:** Alex Chao, Hussein Al-Ghoul, Andrew D. McEachran, Ilya Balabin, Tom Transue, Tommy Cathey, Jarod N. Grossman, Randolph R. Singh, Elin M. Ulrich, Antony J. Williams, Jon R. Sobus

**Affiliations:** 1 Oak Ridge Institute for Science and Education (ORISE) Participant, 109 T.W. Alexander Drive, Research Triangle Park, NC 27711 USA; 2 Student Contractor, U.S. Environmental Protection Agency, Office of Research and Development, National Exposure Research Laboratory, 109 T.W. Alexander Drive, Research Triangle Park, NC 27711 USA; 3 Present Address: Agilent Technologies Inc., Santa Clara, CA 95051 USA; 4 General Dynamics Information Technology, 79 T.W. Alexander Drive, Research Triangle Park, NC 27709 USA; 5 Present Address: Luxembourg Centre for Systems Biomedicine, University of Luxembourg, 4365 Esch-sur-Alzette, Luxembourg; 6 U.S. Environmental Protection Agency, Office of Research and Development, National Exposure Research Laboratory, 109 T.W. Alexander Drive, Research Triangle Park, NC 27711 USA; 7 U.S. Environmental Protection Agency, Office of Research and Development, National Center for Computational Toxicology, 109 T.W. Alexander Drive, Research Triangle Park, NC 27711 USA

**Keywords:** Non-targeted analysis, High-resolution mass spectrometry, CFM-ID, ENTACT, ToxCast, DSSTox

## Abstract

High-resolution mass spectrometry (HRMS) enables rapid chemical annotation via accurate mass measurements and matching of experimentally derived spectra with reference spectra. Reference libraries are generated from chemical standards and are therefore limited in size relative to known chemical space. To address this limitation, in silico spectra (i.e., MS/MS or MS2 spectra), predicted via Competitive Fragmentation Modeling-ID (CFM-ID) algorithms, were generated for compounds within the U.S. Environmental Protection Agency’s (EPA) Distributed Structure-Searchable Toxicity (DSSTox) database (totaling, at the time of analysis, ~ 765,000 substances). Experimental spectra from EPA’s Non-Targeted Analysis Collaborative Trial (ENTACT) mixtures (*n* = 10) were then used to evaluate the performance of the in silico spectra. Overall, MS2 spectra were acquired for 377 unique compounds from the ENTACT mixtures. Approximately 53% of these compounds were correctly identified using a commercial reference library, whereas up to 50% were correctly identified as the top hit using the in silico library. Together, the reference and in silico libraries were able to correctly identify 73% of the 377 ENTACT substances. When using the in silico spectra for candidate filtering, an examination of binary classifiers showed a true positive rate (TPR) of 0.90 associated with false positive rates (FPRs) of 0.10 to 0.85, depending on the sample and method of candidate filtering. Taken together, these findings show the abilities of in silico spectra to correctly identify true positives in complex samples (at rates comparable to those observed with reference spectra), and efficiently filter large numbers of potential false positives from further consideration.

**Electronic supplementary material:**

The online version of this article (10.1007/s00216-019-02351-7) contains supplementary material, which is available to authorized users.

**Figure:**
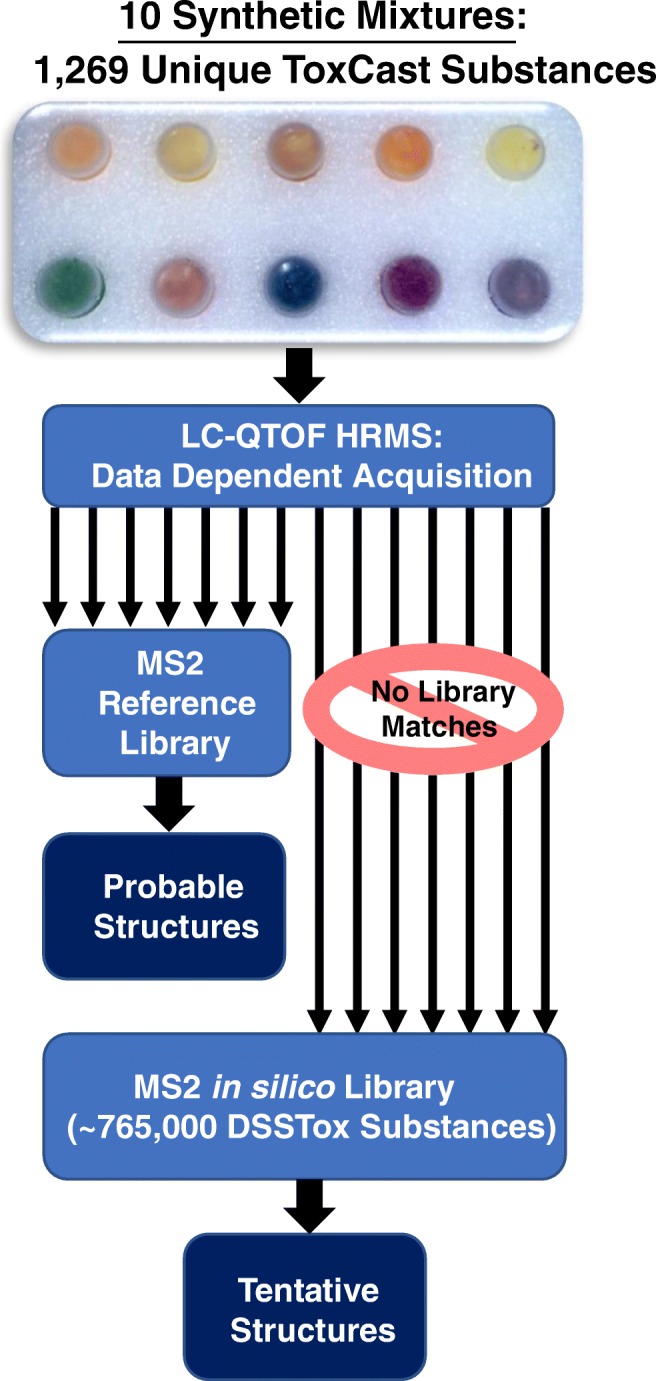
Graphical abstract

## Introduction

The exposome was originally conceived as the sum of all exposures encountered by an individual during their lifetime [1]. Despite more than 10 years of dedicated research, the exposome is not well-characterized for individuals or populations, owing (in part) to a lack of suitable monitoring tools. Traditional exposure monitoring has relied on targeted analytical methods, developed and validated for specific high-interest compounds. These methods have generally proven impractical for exposome studies, where a goal is to characterize previously unknown compounds that may be of eventual interest. Time and resource limitations simply prohibit the development of enough targeted methods to cover the expanse of the exposome.

Advancements in analytical and computational technologies have enabled a shift from targeted monitoring methods to non-targeted analysis (NTA) methods. High-resolution mass spectrometers (HRMS), utilizing Orbitrap and quadrupole time-of-flight (Q-TOF) mass analyzers, now provide the combination of resolution, sensitivity, and speed needed to support NTA studies. Whereas targeted methods only monitor specific compounds during data acquisition, HRMS instruments generate data with sufficient quality that compound selection/identification can be performed at later stages of analysis, without reliance on pre-conceived chemical target lists. The confidence in eventual chemical identifications depends, in part, on the experimental HRMS data available for analysis. Accurate mass and isotope pattern data may enable chemical characterization at the molecular formula level, whereas tandem fragmentation data (i.e., MS/MS or MS2 spectra) may enable characterization at the structure level [2]. Highly confident identifications are generally those in which experimental MS2 data are matched to reference MS2 data contained within a well-curated library (with confirmation ultimately requiring use of a chemical standard). Numerous reference libraries exist (e.g., mzCloud, MassBank, NIST) and enable confident identifications in NTA studies; these range from proprietary vendor-generated libraries to public repositories reflecting the collaborative efforts of many contributors. Recent reviews highlight the breadth of these MS2 reference libraries, which include spectra for up to tens of thousands of compounds [3–5]. Compared with chemical listings within ChemSpider and PubChem (numbering in the millions), however, these libraries cover only a small fraction of potential chemicals of interest [6, 7].

Chemical coverage within reference libraries is unlikely to change dramatically in the near future; the requirement for chemical synthesis followed by MS analysis is rate-limiting in the growth of said libraries. To address this challenge, researchers have turned to computational approaches, wherein computer-generated spectra (or fragment ions) are the basis for comparison against experimental data. Using these in silico approaches, library coverage is limited only by the size of the database from which the predictions are based.

A variety of approaches currently exist for spectra/fragment prediction and comparison. Approaches like MS-Finder and Mass Frontier use specific fragmentation rules to predict MS2 spectra for database compounds [8]. An inherent limitation of this approach is a bias towards compounds for which the known rules apply. Other approaches like MetFrag and MAGMA use combinatorial fragmentation. Here, rather than predicting spectra for a given compound, each bond of that compound is systematically broken in silico to yield possible molecular fragments. Experimental fragment ions are then matched against possible molecular fragment ions to generate a weighted score for that compound [9–11].

Molecular fingerprinting is another computational technique, and is being utilized by ChemDistiller and CSI:FingerID. With this approach, predictive analysis is performed on experimental data [12–14]. Specifically, fragment ions within an experimental spectrum are used to predict specific structural features (i.e., substructures) of the unknown compound, which together yield a “fingerprint” for that compound. The predicted fingerprint for the unknown compound is compared with discrete fingerprints for database compounds to yield a list of scored matches. Recent reviews highlight the merits and limitations of these computational approaches for the analysis of experimental MS2 data [3, 15, 16].

Competitive Fragmentation Modeling-ID (CFM-ID) is an approach wherein experimental MS2 spectra are searched and scored against predicted spectra based on similarity [17, 18]. CFM-ID algorithms are trained on experimental data and used to discover fragmentation rules and eventual predictive models for MS2 spectra. Relative to previously described computational approaches, CFM-ID exists in a middle ground; predicted spectra are more complex than those based on specific fragmentation rules, while avoiding the explosion of fragmentation possibilities from combinatorial methods. CFM-ID further predicts peak intensities, which can be incorporated into spectral similarity searches and match scores. The source code for CFM-ID is publicly available, allowing for incorporation into in-house databases. Predictions can thus be pre-processed on the entirety of a chemical database, reducing computational time during actual searching of experimental data.

With several computational approaches available, numerous performance comparisons have been conducted in recent years [11, 13, 17]. Unsurprisingly, results have varied from assessment to assessment, as the tested data sets have differed from one study to the next. To address this challenge, the Critical Assessment of Small Molecule Identification (CASMI) contest was founded in 2012 with the goal of enabling a more accurate comparison between methods. For each CASMI contest, an MS-based data set of challenge compounds unknown to the participants was made publicly available for examination [19, 20]. Specifically, previously acquired MS2 spectra (with accompanying metadata, in some instances) for individual compounds were shared for blinded evaluation. Results for each completed contest year have been compiled and are available online (http://casmi-contest.org), along with the challenge data sets, allowing for additional testing of new/refined computational approaches.

The data sets and results available through CASMI are an excellent resource for evaluating specific computational tools and in silico libraries. Since the CASMI contests were focused on evaluating spectra of individual compounds, a logical extension is to consider many spectra from a complex mixture as part of a performance evaluation. Along these lines, EPA’s Non-Targeted Analysis Collaborative Trial (ENTACT) was launched in 2016 to evaluate the current status and landscape of NTA approaches, from data acquisition through results processing, with a focus on xenobiotic compounds in complex mixtures [21, 22]. Ten ENTACT mixtures were ultimately prepared, encompassing over 1200 chemical substances from EPA’s Toxicity Forecaster (ToxCast) library, and sent to participating labs for analysis. Much like CASMI, participants were allowed freedom in the selection of NTA approaches. While initially blinded, labs were eventually informed of the contents of each mixture to enable self-evaluation.

Within EPA’s Office of Research and Development (ORD), initial analysis of the ENTACT mixtures has been performed and results of self-evaluation reported [23]. The purpose of the current article is to describe the incorporation of CFM-ID predicted spectra into the existing EPA workflow, and to evaluate overall method performance using the ENTACT mixture data. CFM-ID was selected for this investigation given the availability of the source code and its documented performance in previous CASMI contests. This article describes (1) workflows for processing and searching experimental MS2 spectra against CFM-ID predicted spectra; (2) approaches for utilizing CFM-ID search scores in NTA workflows; (3) assessment of CFM-ID performance on ENTACT mixture compounds; and (4) comparison of reference library performance vs. CFM-ID library performance. This analysis serves as the initial proof-of-concept for adding CFM-ID predictions to an established NTA workflow. Future analyses that utilize this addition will benefit from increased library coverage and enhanced confidence in compound identifications.

## Methods

**Fig. 1:**
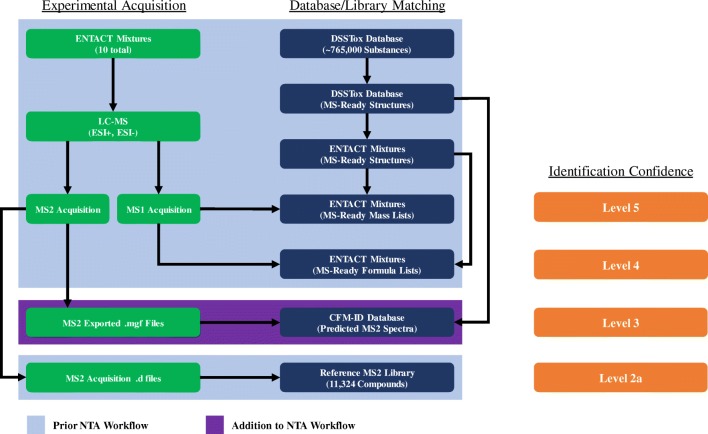
Overall workflow for data acquisition and compound identification. Sections outlined in blue show aspects of the workflow previously implemented for the analysis of ENTACT mixtures. The section outlined in purple shows additions to the workflow that involve matching experimental MS2 spectra with CFM-ID predicted spectra. Identification confidence levels [2] for each match of experimental data to a corresponding database/library entry are shown alongside the specified match in the workflow

Figure 1 displays the overall NTA workflow utilized in our analyses of the ENTACT mixtures. This workflow outlines the main components of data acquisition and processing (left), as well as database generation and matching (center). It further lists the confidence levels associated with each type of match (right). Our previously reported results for the ENTACT mixtures were based on matching feature data to mass lists, formula lists, and reference MS2 libraries (highlighted in blue) [23]. The current examination incorporates searching against CFM-ID predicted spectra (highlighted in purple).

### Sample preparation and data acquisition

Sample preparation and analysis procedures have been previously described [23]. Briefly, a total of 1269 unique substances were spiked across ten separate synthetic mixtures (labelled 499 through 508), with each mixture receiving between 95 and 365 substances. Each mixture was analyzed via liquid chromatography/mass spectrometry (LC/MS), utilizing an Agilent 1290 Infinity II LC coupled to an Agilent 6530B accurate mass quadrupole time-of-flight (Q-TOF) mass spectrometer with a Dual AJS ionization source. An Agilent ZORBAX Eclipse Plus C8 column (2.1 × 50 mm, 1.8 μm) was used along with mobile phases consisting of 0.4 mM ammonium formate buffer in water and methanol. MS1 and MS2 data were collected in a scan range of 100–1000 *m/z* in both positive and negative ionization modes. Reference solution consisting of purine, hexakis(1H,1H,3H-tetrafluoropropoxy)phosphazene, and trifluoroacetic acid (TFA) was infused into the source during the course of the run for auto-correction of mass drift. MS2 data were acquired using Auto MS2 acquisition with the following settings: 3 max precursors per cycle, minimum threshold 3000 counts, scan rate 4 spectra/second. MS2 exclusion lists were generated to exclude ions corresponding to the reference solution from selection for fragmentation. MS2 inclusion lists were generated to increase preference for ions corresponding to substances previously observed using MS1 data. Each sample was acquired three times to generate MS2 data, with each acquisition collecting at one of the three collision energy (CE) levels: 10, 20, or 40 V.

### Chemical substance database

EPA’s Distributed Structure-Searchable Toxicity (DSSTox) Database is a public chemistry resource containing data on (at the time of analysis) ~ 765,000 chemical substances and serves as the foundation for EPA’s CompTox Chemicals Dashboard, hereafter referred to as the Dashboard (https://comptox.epa.gov/dashboard) [24, 25]. Each chemical substance within DSSTox is identified by a unique DSSTox substance identifier (DTXSID) and is also mapped to a “MS-Ready” structure corresponding to the form that would be observed by MS analysis. “MS-Ready” structures are identified by DSSTox chemical identifiers (DTXCID) [26]. The entirety of the 1269 unique ENTACT mixture substances is registered within DSSTox, with unique DTXSIDs and associated MS-Ready DTXCIDs.

### Substance selection for MS2 matching

In a previous analysis of the ENTACT mixtures, initial substance identification was performed without the use of individual reference standards. Thus, for any given spiked substance, determination of presence vs. absence could not be made with absolute certainty (i.e., Schymanski et al. level 1) [23]. Features that could be linked to spiked substances with enough diagnostic evidence (e.g., MS1 and MS2 data corroborating an identification at the “probable structure” level [2]) were classified as “passes,” indicating that there was strong evidence of their presence. The set of “pass” substances, spanning all ten mixtures, was the basis for all analyses in the current study. Specifically, these “pass” substances were first used to generate lists of expected monoisotopic masses, considering only [M+H]^+^ and [M-H]^-^ ion species for positive and negative ESI modes, respectively. These lists of expected masses were then searched (with a 10-ppm accuracy window) against MS2 precursor ion lists to identify “pass” substances for which MS2 data were acquired.

### Reference library preparation

Reference MS2 spectra were contained in Agilent Personal Compound Database and Library (PCDL) format. Six Agilent PCDLs (i.e., Environmental water screening, Pesticides, Forensic toxicology, Veterinary drugs, Metlin, and Extractables and leachables) were combined and used for the current analysis. Experimental MS2 data [23] were searched against the composite PCDL using Agilent MassHunter Qualitative Analysis (version B.08) software with forward and reverse scoring thresholds of 0 and 20, respectively. All matches were manually reviewed to increase confidence in compound identifications.

Compound information from each of the six PCDLs was exported using Agilent PCDL Manager software. Specifically, compound name, formula, mass, CAS number, and number of MS2 spectra were exported for all compounds in each PCDL. This list of compounds was filtered for those containing at least one MS2 spectrum, and then batch searched by CAS number on the Dashboard to retrieve a DTXSID for each compound in the PCDLs. MS-Ready DTXCIDs were then retrieved for each compound by querying a DSSTox MS-Ready mapping file. In some cases, a PCDL compound was not able to be mapped to a DTXSID/DTXCID, either due to the compound not being registered in DSSTox or due to an incorrect CAS number preventing a mapping. PCDL compounds were compared against the ENTACT mixture compounds by MS-Ready DTXCID to estimate the approximate coverage of ENTACT mixture compounds within the searched PCDLs.

### In silico library preparation

In silico MS2 spectra were computed for the majority of MS-Ready structures in DSSTox using the publicly available CFM-ID 2.0 algorithms [17]. Predictions were based on electrospray ionization, in positive and negative modes, at three CE levels (10, 20, and 40 V). Briefly, SMILES strings for MS-Ready structures in DSSTox were input into the CFM-ID prediction source code (http://sourceforge.net/projects/cfm-id) with pre-trained parameters. Resulting predicted spectra were then linked with MS-Ready structure metadata such as DTXCID, molecular formula, and monoisotopic mass. The resulting database of CFM-ID predicted spectra is hereafter referred to as the “CFM-ID database” [27].

### In silico library matching

Fig. S1 (see Electronic Supplementary Material, ESM) describes the workflow for searching ENTACT MS2 spectra against the CFM-ID database (source code used for in silico library matching, scoring, and processing of results is available at https://github.com/NTA-Code/cfmid). Acquired MS2 spectra were first exported from Agilent .d files in MGF format, and then processed using a custom script written in the Python programming language. Processing of MGF files was performed to improve data formatting and to de-duplicate MS2 spectra. Regarding de-duplication, any single chemical feature with an associated precursor mass may generate multiple MS2 spectra during acquisition. The spectrum with the highest signal was considered most representative of the chemical feature for spectral matching purposes. Thus, for a given precursor mass, the spectrum with the highest sum intensity of ions was retained for analysis. Once MS2 spectra were processed, the Python script searched the CFM-ID database for all candidate compounds (as identified by MS-Ready DTXCID) within a 10-ppm mass window of each MS2 spectrum precursor mass, considering only [M+H]^+^ and [M-H]^-^ ion species for positive and negative modes, respectively. The Python script then scored predicted spectra (for CE 10, 20, and 40 V) for all candidates against the experimental MS2 spectrum using a dot-product algorithm [28] with a fragment mass window of 0.02 Da, with scores ranging from 0 to 1.

**Fig. 2:**
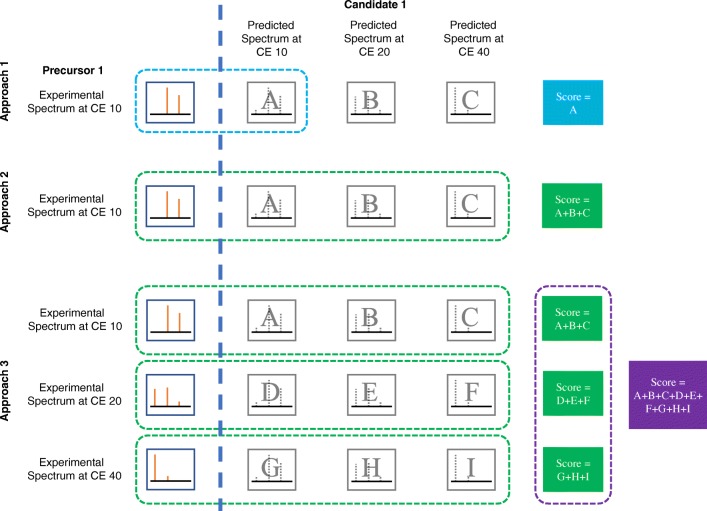
Three approaches for utilizing CFM-ID scores. Each combination of experimental spectrum vs. CFM-ID predicted spectrum generates a unique score via the dot-product algorithm, designated by a unique letter assignment. In approach 1, only one score is generated at the designated collision energy (CE, where CE_experimental_ = CE_in silico_). In approach 2, scores from all three CE_in silico_ levels are summed. In approach 3, scores are summed across all three CE_in silico_ levels, and then across all three CE_experimental_ levels

Once scores were generated for candidate compounds, three approaches for using the scores were evaluated (Fig. 2). In approach 1, only the score of the CFM-ID spectrum with the same CE level as the experimental spectrum was used. In approach 2, scores for CFM-ID spectra at all three CE levels were summed as a new score. In approach 3, scores for CFM-ID spectra at all CE levels were summed as a new score, and these new scores were summed across all experimental CE levels. Scores from each approach were used to rank ENTACT mixture compounds against other candidate compounds for each MS2 spectrum. Scores were also used to generate percentile and quotient values for all candidate compounds, with quotient values defined as the score of the candidate compound divided by the maximum score amongst all candidate compounds for a given experimental MS2 spectrum.

Only MS2 spectra corresponding to “pass” ENTACT mixture compounds were evaluated by CFM-ID library matching. For each MS2 spectrum, the ENTACT mixture compound represents a true positive (TP) and the remaining candidate compounds represent potential false positives (FP). When a cutoff filter is applied to CFM-ID results based on either a percentile or quotient value, the ENTACT mixture compound is considered either a potential TP (if above the cutoff value) or a false negative (FN; if below the cutoff value). Other candidate compounds which are above the cutoff value are considered potential FPs, and those below the cutoff value are considered true negatives (TN). Examples of cutoff filtering of CFM-ID results are shown in Fig. S2 (see ESM). True positive rates (TPRs) and false positive rates (FPRs) were calculated using the following equations:

$$ \mathrm{TPR}=\frac{\mathrm{TP}}{\mathrm{TP}+\mathrm{FN}} $$

$$ \mathrm{FPR}=\frac{\mathrm{FP}}{\mathrm{FP}+\mathrm{TN}} $$

To identify an optimal threshold for candidate filtering, cutoff values were incremented throughout the entire range by hundredths of the value range (i.e., percentile cutoffs were set to 0, 1, 2 … 100; quotient cutoffs were set to 0, 0.01, 0.02 … 1). At each level, TP, FP, TN, and FP counts were tallied and used to calculate TPR and FPR. Receiver operating characteristic (ROC) curves were then generated, using TPR and FPR values, for the global ENTACT data set (i.e., all ten mixtures). Using the global curves, the percentile value and quotient value that would result in a minimum TPR of 0.90 were determined. These global percentile and quotient cutoffs were applied to each ENTACT mixture’s results to calculate the mixture-specific TPR and FPR based on the global cutoff. The mixture-specific TPRs and FPRs ultimately serve as performance metrics for the proposed methods.

Some NTA workflows base predicted library matching on monoisotopic mass queries, whereas others restrict the candidate compound set to those matching a specific formula (deduced from MS1 spectra or other orthogonal methods). All procedures described in the “In silico library matching” section were performed separately based either on monoisotopic mass queries or on mass queries followed by formula filtering (where the MS-Ready formula of all candidates was forced to match that of the “pass” substance). It is noteworthy that, for this investigation of ENTACT mixtures, a single formula was previously assigned to each “pass” substance with a high level of confidence. Formula assignments for features in true unknown samples are subject to considerably larger error rates. Thus, results of our formula-based analysis represent a “best case scenario” and yield the smallest expected FPRs. Nevertheless, comparison of results based on mass vs. formula queries will help establish best practices and performance targets for predicted library matching protocols.

## Results

### Reference library matching

For a given ENTACT compound, identification via reference library matching requires that the compound is ionizable (given the experimental source conditions), selected for MS2 acquisition, and present in the reference library. As described above, our previous analysis of the ENTACT mixtures yielded a list of “pass” substances that were identified with sufficient diagnostic evidence; this list of substances (ESM Table S1) represents the starting point for the current evaluation. It is noteworthy that certain substances were included in multiple mixtures as part of the ENTACT design to help evaluate method reproducibility [21, 23]. For the purposes of this analysis, the focus of which was to evaluate performance of in silico library matching across a broad range of substances, each substance was ultimately evaluated only once even if it was acquired in multiple mixtures. Initial results (vide infra), however, are provided without de-duplication to preserve statistics specific to each individual ENTACT mixture.

Overall, 44% of spiked ENTACT substances were classified with a “pass” rating (Table 1). Certain ENTACT mixtures (e.g., 507 and 508) had a very low proportion of “pass” compounds owing, in part, to a high number of spiked isomers that could not be resolved even with MS2 data. Out of 845 total “pass” compounds, 500 (59%) were included in the composite PCDL (including reference MS2 data), 453 (54%) had acquired MS2 data, and 300 (36%) had both reference and acquired MS2 data (Table 1). Ultimately, 246 of these 300 “pass” compounds were correctly identified with a level 2a designation [2]. Thus, an 82% success rate was observed when considering “pass” compounds with both experimental and reference MS2 data (*n* = 300). A 54% success rate, however, was observed when considering all “pass” compounds with experimental MS2 data (*n* = 453), regardless of whether they were in the composite PCDL.

**Table 1:** Numbers of spiked ENTACT substances meeting specific research criteria

Mixture	Spiked substances	Passes	Passes in PCDL^1^	Passes w/ MS2	Passes in PCDL and w/ MS2	Passes matched by PCDL
499	95	46	28	37	23	18
500	95	19	14	14	11	7
501	95	47	28	34	25	23
502	95	58	42	22	17	15
503	185	103	59	67	43	34
504	185	103	55	68	41	34
505	365	224	128	64	44	40
506	365	195	114	113	74	61
507	95	19	13	14	9	7
508	364	31	19	20	13	7
Total	1939	845	500	453	300	246
% of total	NA	44%	26%	23%	15%	13%
% of passes	NA	NA	59%	54%	36%	29%

^1^Composite “Personal Compound Database and Library” (PCDL) containing compounds from six individual Agilent PCDLs (i.e., Environmental water screening, Pesticides, Forensic toxicology, Veterinary drugs, Metlin, and Extractable and leachables)

### In silico library matching

#### Evaluation by collision energy

Regarding the use of in silico spectra for compound identification, initial goals of this evaluation were to determine whether 1:1 matching (i.e., one experimental spectrum vs. one in silico spectrum) is best performed at a common CE level, and whether a specific CE level (10, 20, or 40 V data) would stand out as yielding the best results. To achieve these goals, MS2 spectra for “pass” compounds were scored against their respective CFM-ID spectra at all three CE levels. As shown in Fig. S3 (see ESM), the highest match scores (where CE_experimental_ = CE_in silico_) were generally observed at a CE of 10 V, followed by those observed at 20 V and 40 V. These results likely reflect (1) the presence and matching of intact precursor ions at lower CE levels and (2) greater spectral complexity and number of fragments (with some below the experimental mass range) at higher CE levels.

Fig. S4 (see ESM) shows, at each CE_experimental_ for each “pass” compound, the quotient of the CFM-ID score when CE_experimental_ = CE_in silico_ vs. the CFM-ID score when CE_experimental_ ≠ CE_in silico_. For each comparison group (*n* = 6), the estimated median value was significantly greater than 1 (Wilcoxon signed-rank test; *p* < 0.0001 in all cases), reflecting higher CFM-ID scores when CE_experimental_ = CE_in silico_. Not surprisingly, median quotients were highest when the CE_experimental_ and CE_in silico_ were most dissimilar (e.g., 10V_score_/40V_score_). Examination of the range of quotients shows that, for some “pass” compounds, the CFM-ID scores were over 1000 times higher when CE_experimental_ = CE_in silico_ vs. when CE_experimental_ ≠ CE_in silico_. In other cases, however, the CFM-ID scores were up to 100 times lower when CE_experimental_ = CE_in silico_. These results highlight the potential value in utilizing in silico spectra at non-matching CE levels as part of a composite score. The value of such a proposition is examined below via scoring approaches 2 and 3.

#### Evaluation by scoring method

Three different scoring approaches were compared (Fig. 2), with scores based on (1) 1:1 matching between experimental and in silico spectra (where CE_experimental_ = CE_in silico_); (2) 1:3 matching with summation across three CFM-ID match scores for a given experimental spectrum; and (3) summation of scores across all possible combinations (*n* = 9) of experimental vs. in silico spectra. Each approach was evaluated for all “pass” compounds across all ten ENTACT mixtures.

Distributions of ranks for “pass” compounds amongst all candidate compounds retrieved from the CFM-ID database are given in Table 2 (without formula filtering) and Table 3 (with formula filtering). For approaches 1 and 2, the best results were observed when CE_experimental_ = 20 V. Results using approach 3 were very comparable to the best results from approaches 1 and 2. Overall, when database matching was performed without formula filtering (Table 2), the spiked compound was ranked as the top candidate up to 38% of the time, within the top 5 candidates up to 60% of the time, and within the top 20 candidates up to 79% of the time. Using approach 3, the spiked compound ranked in the 81st percentile of all candidate compounds, on average, when considering CFM-ID match scores.

**Table 2:** CFM-ID results for ENTACT mixture compounds across three scoring approaches (Fig. 2). Candidate compounds from the CFM-ID database were limited to those having an MS-Ready monoisotopic mass matching (within 10 ppm) that of the known (spiked) substance

	Approach 1			Approach 2			Approach 3
CE_experimental_	10	20	40	10	20	40	Σ^a^
CE_in silico_	10	20	40	Σ	Σ	Σ	Σ
No. of compounds scored	363	368	360	363	368	360	377
Number of true positives							
Top hit	102	129	93	100	139	100	129
Within top 5	187	219	162	188	221	162	224
Within top 20	267	279	215	275	283	213	298
Percentage of true positives							
Top hit	28%	35%	26%	28%	38%	28%	34%
Within top 5	52%	60%	45%	52%	60%	45%	59%
Within top 20	74%	76%	60%	76%	77%	59%	79%
Average percentile for true positives	77th	81st	72nd	78th	82nd	73rd	81st
Average quotient for true positives	0.67	0.62	0.45	0.64	0.65	0.47	0.69

^a^Sum of three CEs

**Table 3:** CFM-ID results for ENTACT mixture compounds across three scoring approaches (Fig. 2). Candidate compounds from the CFM-ID database were limited to those having an MS-Ready formula matching that of the known (spiked) substance

	Approach 1			Approach 2			Approach 3
CE_experimental_	10	20	40	10	20	40	Σ^a^
CE_in silico_	10	20	40	Σ	Σ	Σ	Σ
No. of compounds scored	363	368	360	363	368	360	377
Number of true positives							
Top hit	159	178	123	171	180	128	188
Within top 5	239	250	194	243	252	194	268
Within top 20	284	291	232	295	292	232	321
Percentage of true positives							
Top hit	44%	48%	34%	47%	49%	36%	50%
Within top 5	66%	68%	54%	67%	68%	54%	71%
Within top 20	78%	79%	64%	81%	79%	64%	85%
Average percentile for true positives	82nd	83rd	76th	83rd	84th	77th	84th
Average quotient for true positives	0.77	0.73	0.57	0.77	0.75	0.59	0.79

^a^Sum of three CEs

As expected, results were markedly better, regardless of the scoring approach, when implementing formula filtering as part of candidate ranking (Table 3). Again, results for approach 3 were very similar to those for approaches 1 and 2 when CE_experimental_ = 20 V. This time, however, the spiked compound was ranked as the top candidate up to 50% of the time, within the top 5 candidates up to 71% of the time, and within the top 20 candidates up to 85% of the time. On average, using approach 3, the spiked compound was in the 84th percentile of all candidate CFM-ID match scores. Individual results for each “pass” compound (without and with formula filtering), including the CFM-ID rank of the TP along with number of total candidate compounds, are shown in Fig. S5 (see ESM).

**Fig. 3:**
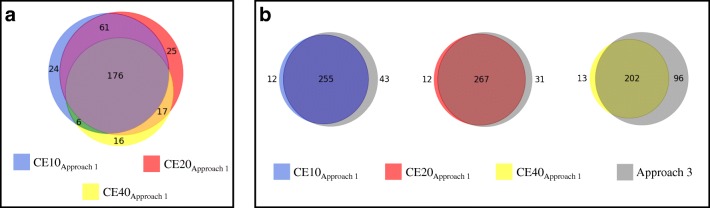
Number of “pass” compounds within the top 20 CFM-ID hits using approach 1 at CE = 10 V vs. 20 V vs. 40 V (**a**). Number of “pass” compounds within the top 20 CFM-ID hits using approach 3 vs. approach 1 at CE = 10, 20, or 40 V (**b**)

Regarding approaches 1 and 2, where a single experimental spectrum is considered at one defined CE_experimental_, performance results generally favor the use of CE = 20 V (Tables 2 and 3). A comparative analysis for approach 1, however, shows benefit of considering all three CE results (Fig. 3a). Specifically, out of 325 unique compounds identified (without formula filtering) as being within the top 20 CFM-ID hits (at one or more CE), 279 were identified at CE = 20 V and 46 were not identified at CE = 20 V (Fig. 3a). Using approach 3, 298 unique compounds were correctly identified as being within the top 20 CFM-ID hits. Approach 3 coverage exceeded that of approach 1 by 31 compounds when CE = 10 V, 19 compounds when CE = 20 V, and 83 compounds when CE = 40 V (Fig. 3b). Considering these findings, composite scoring via approach 3 was used for all remaining evaluations of in silico MS2 spectra.

#### Evaluation of filtering criteria

**Fig. 4:**
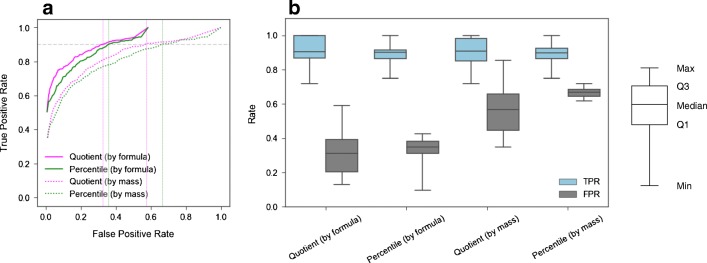
ROC curves (**a**) for ENTACT mixture data (all “pass” compounds from all ten mixtures) when using percentile and quotient cutoff values, and when filtering the CFM-ID database matches by mass or molecular formula. A global TPR of 0.90 (horizontal gray dashed line) results in percentile-based FPR values (green vertical dotted lines) of 0.67 (by mass) and 0.36 (by formula), and quotient-based FPR values (pink vertical dotted lines) of 0.57 (by mass) and 0.32 (by formula). Distributions (**b**) of true positive rates (TPRs) and false positive rates (FPRs) across individual ENTACT mixtures (*n* = 10) when selecting cutoff values based on a global TPR of 0.90 (from **a**)

ROC curves in Fig. 4a show relationships between TPRs and FPRs, at various percentile and quotient cut-points, when candidates from the CFM-ID database were matched to experimental spectra using precursor mass or predicted formula. In general, results based on quotient cutoffs (in pink) are superior to those based on percentile cutoffs (in green). That is, a lower FPR is associated with a given TPR when using a quotient cutoff at a pre-defined test increment. This result is a function of the right-skewed distribution of quotient values vs. the uniform distribution of percentile values (ESM Fig. S6). As expected, results based on formula matching (solid) are superior to those based on precursor mass matching (dotted). This result reflects the smaller number of candidate compounds when implementing a formula filter.

As shown in Fig. 4a, a global TPR of 0.90 (horizontal gray dashed line) yielded percentile-based FPRs (green vertical dotted lines) of 0.67 (by mass) and 0.36 (by formula), and quotient-based FPRs (pink vertical dotted lines) of 0.57 (by mass) and 0.32 (by formula). This global TPR of 0.90 is associated with percentile cutoff values of 32 (by mass) and 38 (by formula), and quotient cutoff values of 0.13 (by mass) and 0.18 (by formula). Figure 4b shows distributions of TPR and FPR values for individual ENTACT mixtures based on these four cutoff values; these distributions highlight expected ranges of TPRs and FPRs when using the CFM-ID database to investigate unknowns in individual samples. Overall, individual mixture TPRs ranged from 0.72 to 1.0, and FPRs ranged from 0.10 to 0.85. Interestingly, more variability in FPRs was observed in analyses utilizing quotient cutoffs. Thus, FPRs are generally expected to be lower, on average, using quotient cutoffs, but more consistent using percentile cutoffs.

### Comparison of performance across reference and in silico libraries

**Fig. 5:**
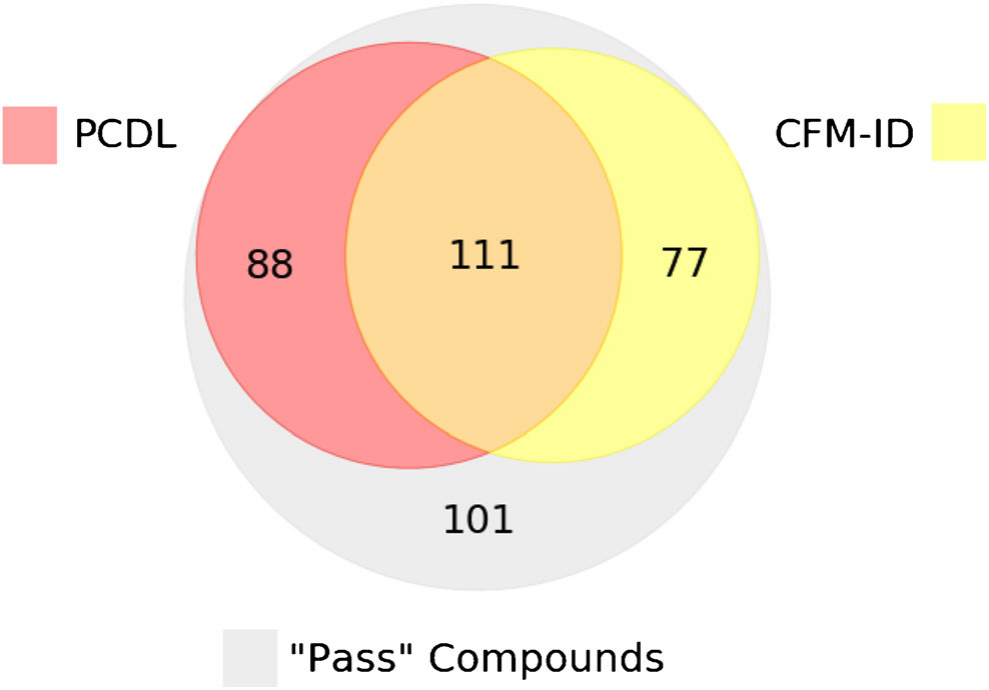
Comparison of “pass” compounds (*n* = 377) correctly identified by reference library matching (using a composite Agilent PCDL) vs. CFM-ID database matching (when filtering by molecular formula)

Figure 5 shows a comparison of de-duplicated “pass” compounds (*n* = 377) that were correctly identified by PCDL reference library matching (*n* = 199) vs. CFM-ID database matching (with formula filtering, *n* = 188). When considering only the top hit from library matching, 88 compounds (23%) were identified only using the composite PCDL, 111 compounds (29%) were identified using both the composite PCDL and the CFM-ID database, and 77 compounds (20%) were identified using only the CFM-ID database. One hundred one (27%) compounds were not identified as the top hit using either the composite PCDL or the CFM-ID database. Ultimately, 53% of “pass” substances were correctly identified by the composite PCDL, and 50% were correctly identified as the top hit using the CFM-ID database. Percentile and quotient-based cutoffs can be used to increase the potential TPR (up to 100%), but at the expense of increasing FPR, as described above. The implementation of cutoff values is at the discretion of the investigator, who must carefully consider the overall objectives of the research study when deciding on a selection strategy.

## Discussion

Targeted methods have long been the gold standard for chemical analysis. As such, they have been implemented in a wide number of scientific fields where chemical detection and/or quantitation is critical. The focused nature of targeted analytical methods has proven limiting in discovery research fields, where chemicals of eventual interest may not yet be known. NTA methods seek to address this shortcoming by enabling discovery and identification of unknown chemicals and informing follow-up targeted investigations.

Confidence in chemical identifications is a function of the experimental information available [2]. As the amount of information supporting an identification increases, the ambiguity surrounding that identification decreases, resulting in more confident annotations. Targeted methods produce data at the highest confidence level, as they utilize chemical standards for which reference MS1, MS2, and chromatographic data can be acquired. NTA methods can benefit from these reference data to the extent that they have been previously acquired and stored in a usable format. Six Agilent PCDLs were used in this analysis as the source of reference MS2 data for matching; the composite of these PCDLs included 11,324 unique compounds with reference MS2 spectra. The ten ENTACT mixtures contained a total of 1269 unique substances, of which 610 (48%) were contained within the composite PCDL. The other 52% of compounds represent a “blind spot” in the reference libraries searched. Clearly, in silico predicted spectra are needed to enable MS2 matching for compounds not captured in empirical libraries. At the time of analysis, CFM-ID predicted spectra were available for ~ 765,000 unique DSSTox compounds, representing a > 60-fold increase in search space over the composite PCDL. Given the obvious advantage of size, careful evaluation of performance is required to ensure proper use and maximum benefit of these predicted spectra.

Experimental MS2 data for ENTACT mixture compounds were collected and CFM-ID spectra predicted at three CE levels (10, 20, and 40 V). The specificity of CE level when matching experimental and predicted spectra was evaluated across all ten ENTACT mixtures. The highest CFM-ID scores were observed when CE_experimental_ = CE_in silico_ (ESM Fig. S4). Furthermore, the best performance, in terms of compound ranking, was generally observed when CE = 20 V (Tables 2 and 3). For some compounds, however, it was more advantageous to acquire and match spectra at CE = 10 or 40 V (Fig. 3a). This is most likely due to variability in compound lability, where different compounds have distinct optimal CE levels needed to generate a spectrum with fragment ions in high abundance. For an NTA workflow where the compounds are unknown, the recommended practice is to acquire experimental MS2 data at all three CE levels in order to capture suitable spectra on the widest range of compounds.

It is difficult to anticipate, for a given compound of interest, whether scoring/ranking results at one CE should be preferred over another. Thus, aggregated scoring approaches were evaluated wherein summed scores were considered across multiple CEs (Fig. 2). It was generally observed that the quality of matching results increased with the amount of data considered, in terms of both experimental and predicted spectra. Specifically, scoring results from approaches 2 and 3 were shown to surpass those from approach 1 at each individual CE (Tables 2 and 3, and Fig. 3b). Approach 3 tended to yield the best overall results and was therefore the basis for performance evaluations regarding TPR and FPR. Moving forward, when using the CFM-ID database as a screening-level tool, we recommend an aggregated approach wherein each experimental spectrum is compared with all three CE levels of predicted spectra (i.e., approach 3).

Utilizing CFM-ID results from approach 3 (based on mass matching (2)), 34% of the 377 ENTACT mixture compounds were identified as the best matching compound. This result is comparable to those reported from the 2016 CASMI contest, in which 12 to 34% of correct candidates were identified as the best matching compound [20]. In certain cases, sub-optimal performance of CFM-ID may reflect dissimilarities in structures between compounds used to train CFM-ID and those included in ENTACT [27]. A re-training of the CFM-ID models with an expanded set of compounds has the potential to improve scoring and ranking results for the ENTACT mixture compounds. Future work will examine the extent to which re-trained models can better identify ENTACT compounds (and potentially other xenobiotics) amongst other candidate chemicals.

Reference libraries are created from empirical spectra and generally yield matches with high accuracy. That is, the best match from a reference library search is often the TP. Predicted libraries are less accurate and, as such, do not always correctly identify the TP as having the best match score. Utilizing results from in silico library searching is therefore a balance between TPR and FPR. Considering only the highest matching compounds will limit the number of FPs, but at a greater risk of missing a TP. A less-stringent cutoff allows for more potential FPs, and also a higher likelihood of retaining the TP. The cutoff threshold depends on the desired goal(s) of the analysis, whether retaining true compounds or eliminating false compounds is of most importance. For this analysis, cutoffs based on percentiles and quotients were evaluated, with candidate selection based on mass matching, with or without additional formula filtering. Our results show a preference for quotient-based cutoffs, and for filtering candidate lists based on molecular formula (Fig. 4a). Specifically, the lowest FPR is expected for a given TPR when using a quotient-based cutoff and formula filtering. Better performance using quotient values is attributed to the skewed (i.e., right-tailed) distribution of quotient values (vs. the uniform distribution of percentile values), where most candidates have very low CFM-ID match scores, and fewer have moderate to high scores (ESM Fig. S6). This allows for more incorrect candidates to be correctly removed from consideration at even a modest cut-point. Interestingly, wider distributions of FPRs were observed when using quotient-based cutoffs vs. percentile-based cutoffs (Fig. 4b). This again stems from the skewed distributions of quotient values and underscores the variable nature of FPRs when using quotient cutoffs. More stable FPRs can be achieved with percentile-based cutoffs; these FPRs are expected to be higher, however, when aiming for a high TPR (~ 0.90).

In silico library matches are inherently less confident than reference library matches. As such, in silico MS2 libraries are not meant to replace reference libraries, but to enable supplementary matching procedures [3, 16, 29]. Figure 5 shows that, using either the reference library (composite PCDL) or the in silico library (CFM-ID database), about half of the “pass” compounds could be correctly identified as the top match. Using both libraries, however, yielded 73% correct identifications. A hybrid approach is therefore highly desirable for the most comprehensive and accurate analysis. For example, in a hypothetical study, MS2 spectra could be matched to both the reference and in silico libraries. Top matches based on the reference library would not require additional support from in silico match scores. Yet, these in silico match scores could serve as the basis for quotient- or percentile-based cut-points. These cut-points would then be used to filter unlikely candidates retrieved from the CFM-ID database. The use of additional supporting information, such as retention time predictions [30, 31] and metadata source counts [20, 32], has been shown to improve NTA identifications; incorporation of these data with CFM-ID ranking results could further improve candidate filtering, thus increasing the overall accuracy and performance of the workflow. Future investigations will aim to incorporate these various data streams into a unified workflow, and to optimize filtering criteria for maximum TPRs and minimum FPRs.

Since the time of this original analysis, EPA’s DSSTox database has increased from ~ 765,000 to ~ 875,000 unique substances; CFM-ID predictions have been generated for the majority of these substances based on their associated “MS-Ready” structures. The dynamic nature of in silico libraries is a highly desirable feature when compared with reference libraries, which are relatively static due to the need for pure standards. This dependence on standards is a significant drawback when investigating new and rapidly emerging chemicals of concern, as the analyses are not able to keep up with the analytes. In silico libraries can be generated at a much more rapid pace, on both known and predicted structures (e.g., those of expected metabolites and transformation products) within a given database. EPA’s DSSTox database is freely available to the public via the Dashboard (https://comptox.epa.gov/dashboard) [24]. Future Dashboard development will provide additional functionality to support HRMS-based NTA workflows (i.e., retention time predictions, media occurrence data, experimental substructure filtering). Updates to the CFM-ID processing and searching workflow are also being explored, including aggregation of multiple experimental spectra into a single spectrum (rather than selecting only the spectrum of highest sum ion intensity), and implementation of intensity threshold filters (for experimental and predicted spectra) prior to CFM-ID matching/scoring. A prototype web-based tool for searching an experimental spectrum against the CFM-ID database has been developed and is undergoing testing; users will see both the candidate results returned for the spectrum as well as visualizations of the predicted vs. experimental spectrum (ESM Fig. S7). CFM-ID batch searching is also being incorporated into existing NTA workflows, with plans to publicly release a stand-alone web service for processing of NTA data. Finally, implementation of CFM-ID 3.0 algorithms (not available at the start of the current project) will likely result in enhanced performance based on an improved in silico library [33].

## Conclusions

Confident identification of unknowns in NTA studies often requires the use of reference library spectra. The relatively modest size of existing reference libraries limits the number of possible identifications for any given study. Use of in silico fragmentation libraries can expand coverage into areas not reached by reference libraries alone. Analyses of the ENTACT mixture data show promising results for the performance of in silico spectra towards aiding chemical identification strategies. The expansion of NTA workflows to incorporate in silico spectra for > 800K DSSTox compounds will enable more rapid and certain identifications of xenobiotics and other emerging compounds.

## Electronic supplementary material

ESM 1 (PDF 42 kb)

ESM 2 (PDF 1.69 mb)

ESM 3 (XLSX 138 kb)
