# Supplementary material for: In silico MS/MS spectra for identifying unknowns: a critical examination using CFM-ID algorithms and ENTACT mixture samples
Source: Anal Bioanal Chem. 2020 Jan 22;412(6):1303–15. doi: 10.1007/s00216-019-02351-7 (PMC7021669; doi:10.1007/s00216-019-02351-7)
Supplement: Supplementary file 1 — (PDF 42 kb) [file 216_2019_2351_MOESM1_ESM.pdf]

Analytical and Bioanalytical Chemistry

Electronic Supplementary Material

In silico MS/MS spectra for identifying unknowns: a critical examination using CFM-ID algorithms and ENTACT mixture samples

Alex Chao, Hussein Al-Ghoul, Andrew D. McEachran, Ilya Balabin, Tom Transue, Tommy Cathey, Jarod N. Grossman, Randolph Singh, Elin M. Ulrich, Antony J. Williams, Jon R. Sobus

Additional files available under 10.1007/s00216-019-02351-7
