# Supplementary material for: In silico MS/MS spectra for identifying unknowns: a critical examination using CFM-ID algorithms and ENTACT mixture samples
Source: Anal Bioanal Chem. 2020 Jan 22;412(6):1303–15. doi: 10.1007/s00216-019-02351-7 (PMC7021669; doi:10.1007/s00216-019-02351-7)
Supplement: Supplementary file 2 — (PDF 1.69 mb) [file 216_2019_2351_MOESM2_ESM.pdf]

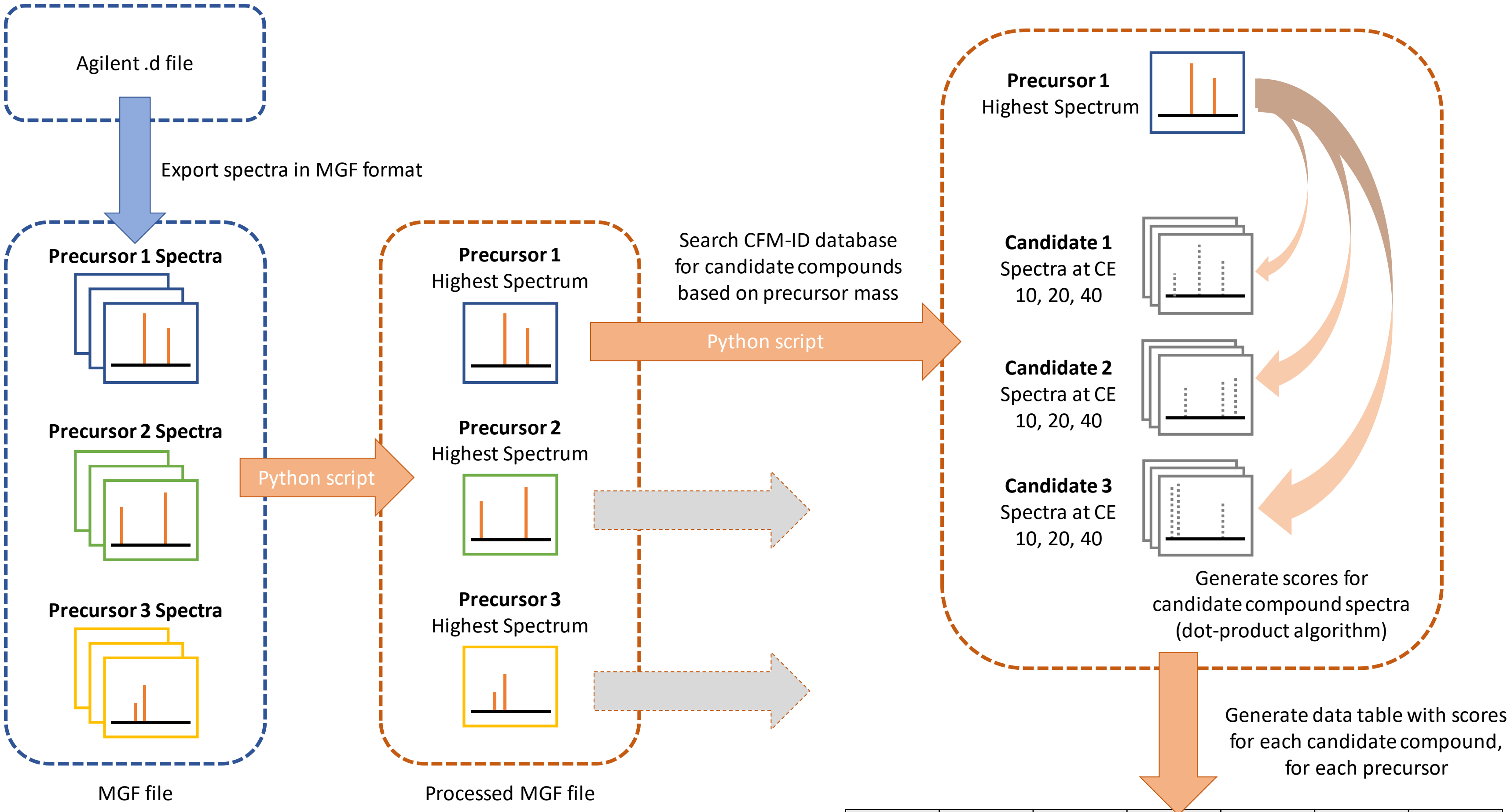

Precursor #	Precursor mass	Candidate name	Candidate mass	Score for CE10 spectrum	Score for CE20 spectrum	Score for CE40 spectrum
Precursor 1	Precursor 1 mass	Candidate 1	Candidate 1 mass	0.5	0.3	0.1
		Candidate 2	Candidate 2 mass	0.1	0.05	0.01
		Candidate 3	Candidate 3 mass	0.2	0.1	0.02
Precursor 2	Precursor 2 mass	Candidate 1	Candidate 1 mass	0.4	0.2	0.1
...	...	...	...	...		

Figure S1. Workflow for parsing experimental (MS2) spectra and matching to CFM-ID predicted spectra. Blue outlines indicate vendor software steps and orange outlines indicate steps taken via custom Python scripts

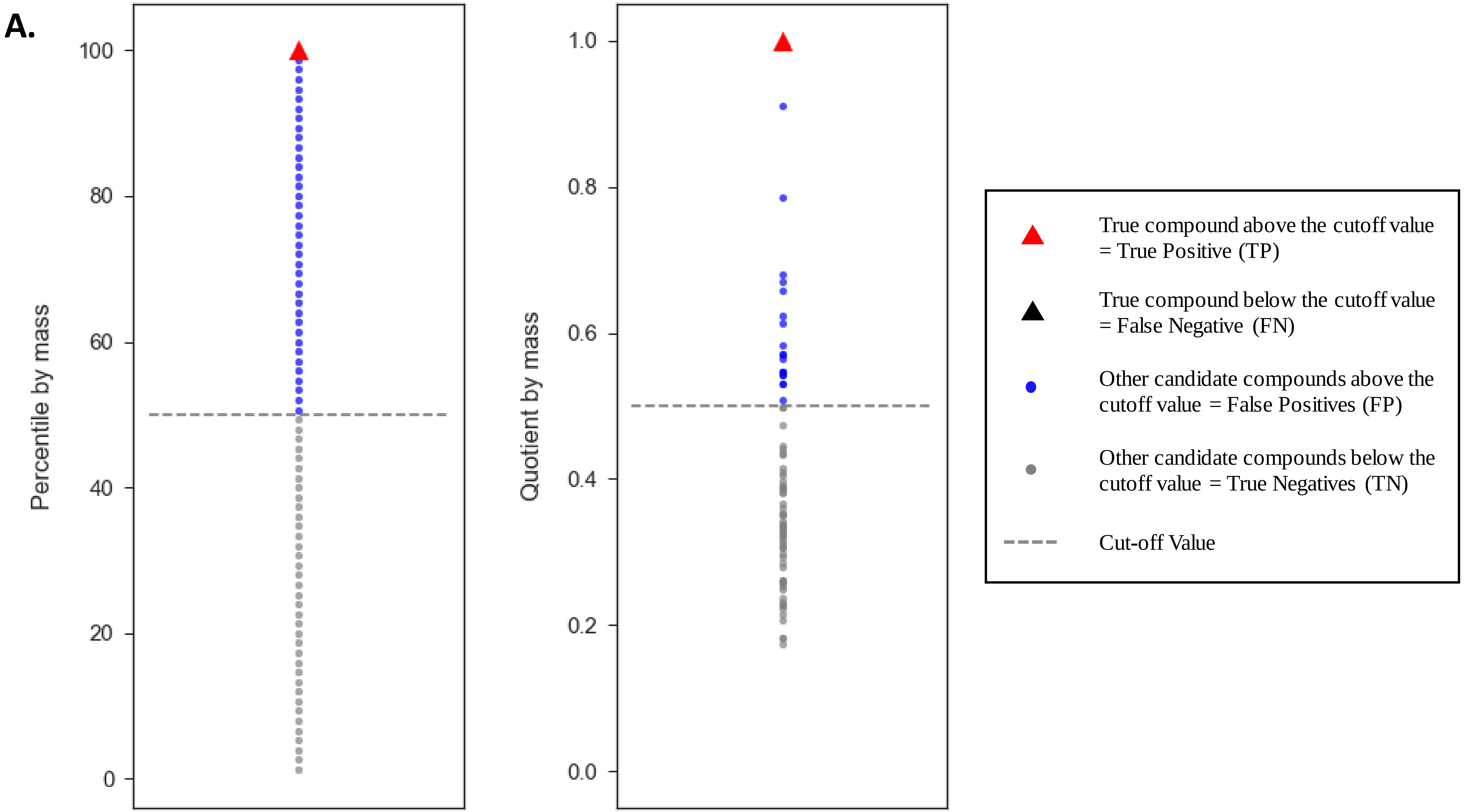

Figure S2A. Distributions of candidate compounds (n=75) for a single CFM-ID query (by mass) based on calculated percentile (left) and quotient (right) values. Here, the "pass" compound was correctly kept above the selected cut-off values, resulting in a "True Positive" (TP) assignment

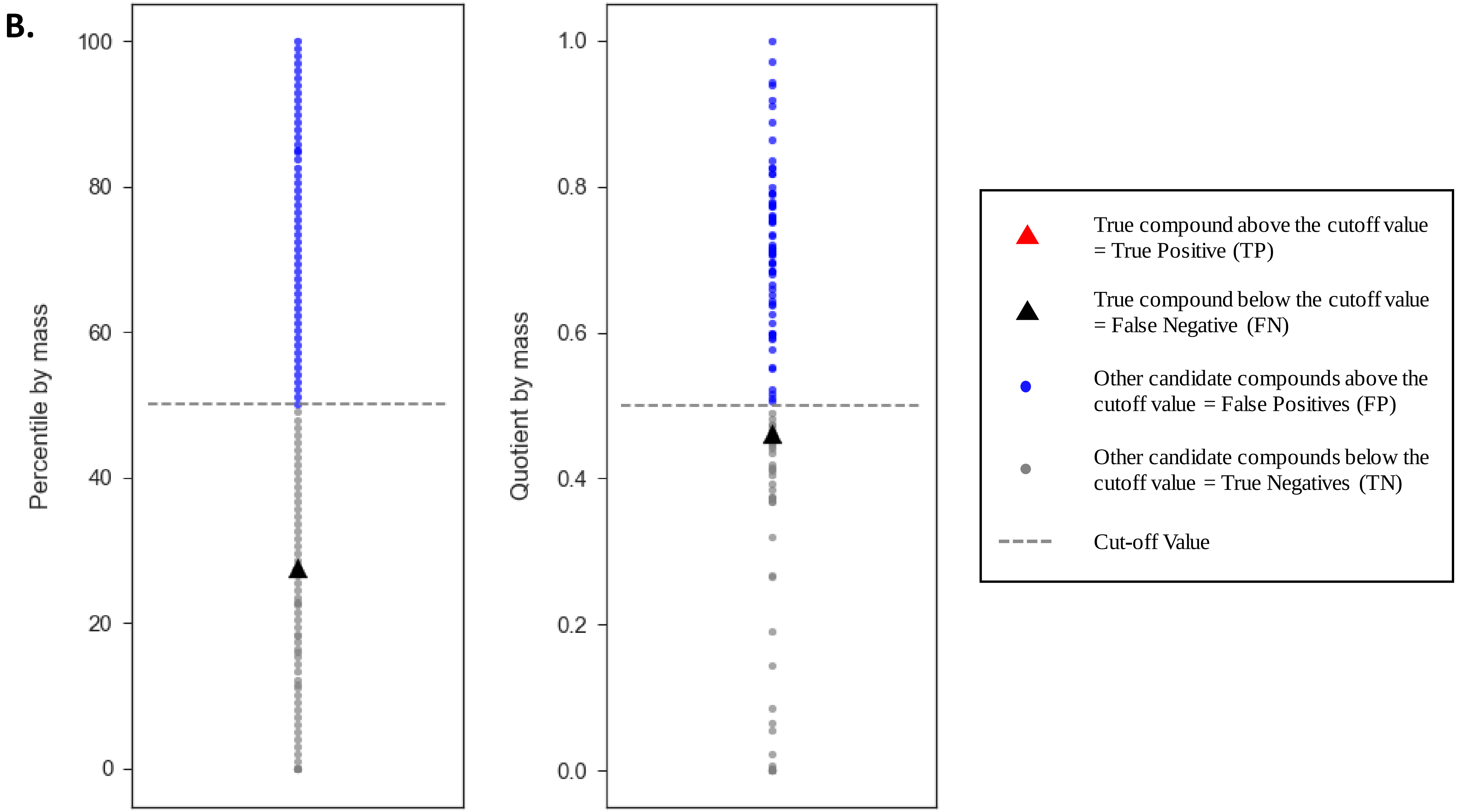

Figure S2B. Distributions of candidate compounds (n=111) for a single CFM-ID query (by mass) based on calculated percentile (left) and quotient (right) values. Here, the "pass" compound was not kept above the selected cut-off values, resulting in a "False Negative" (FN) assignment

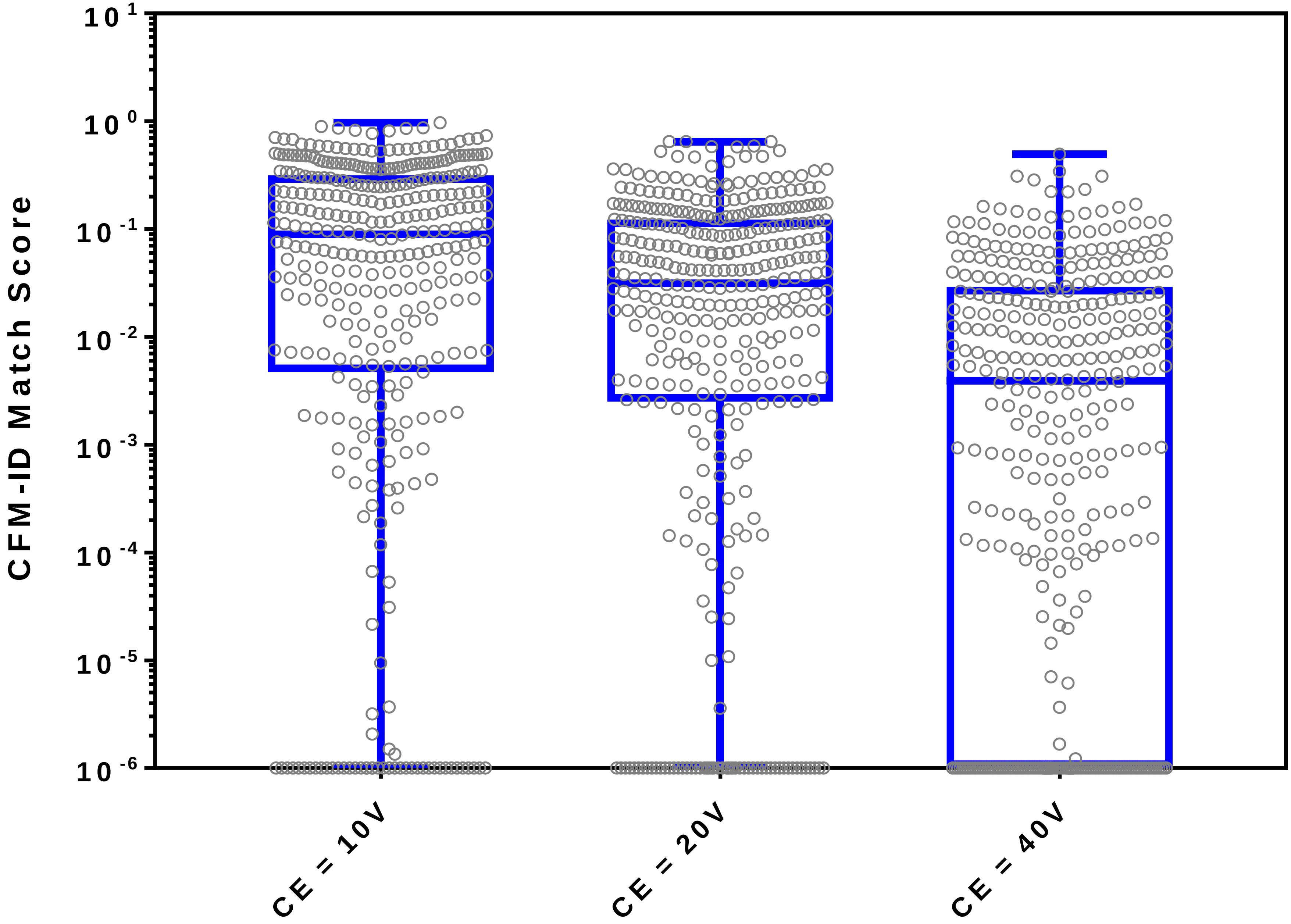

Figure S3. Distributions of CFM-ID match scores at CE=10, 20 and 40V, where $CE_{experimental} = CE_{in\ silico}$

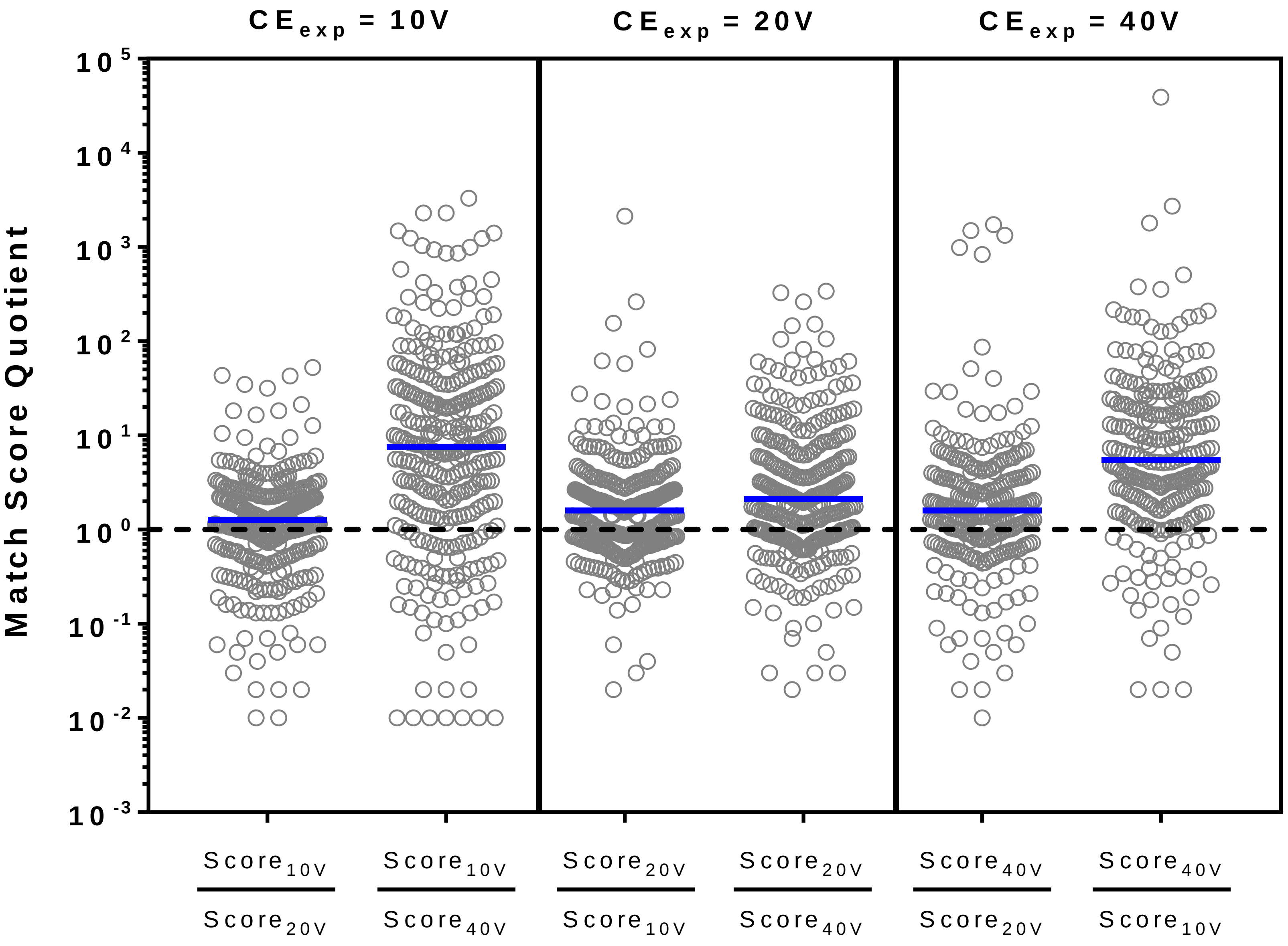

Figure S4. CFM-ID match score quotients for "pass" compounds with MS2 spectra acquired at CE=10, 20 or 40V. Each open circle represents, for a given "pass" compound, the quotient of the CFM-ID score when CE$_{experimental}$ = CE$_{\textit{in silico}}$ vs. the CFM-ID score when CE$_{experimental}$ ≠ CE$_{\textit{in silico}}$. The blue horizontal lines represent the median match score quotients for the individual comparison groups (n=6). For each group, the median match score quotient was significantly greater than 1 (p<0.0001)

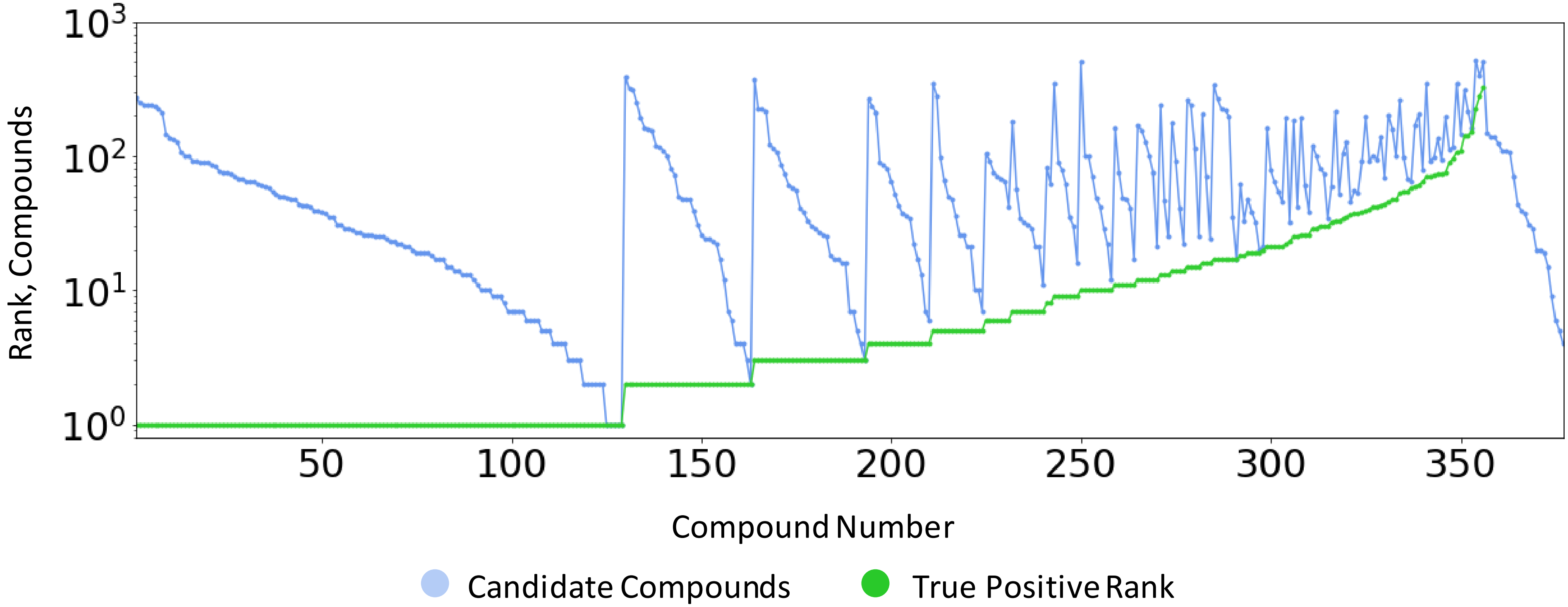

Figure S5A: Results of CFM-ID scoring for all "pass" compounds (n=377) when queried by mass. For each vertical pair, the blue point represents the number of retrieved candidate compounds for a given mass match, and the green point represents the rank of the True Positive (TP) compound. Data are sorted by rank (increasing) and then number of candidate compounds (decreasing). "Pass" compounds without an associated scoring rank had insufficient fragment matches between the experimental and predicted spectra, and therefore, no match score

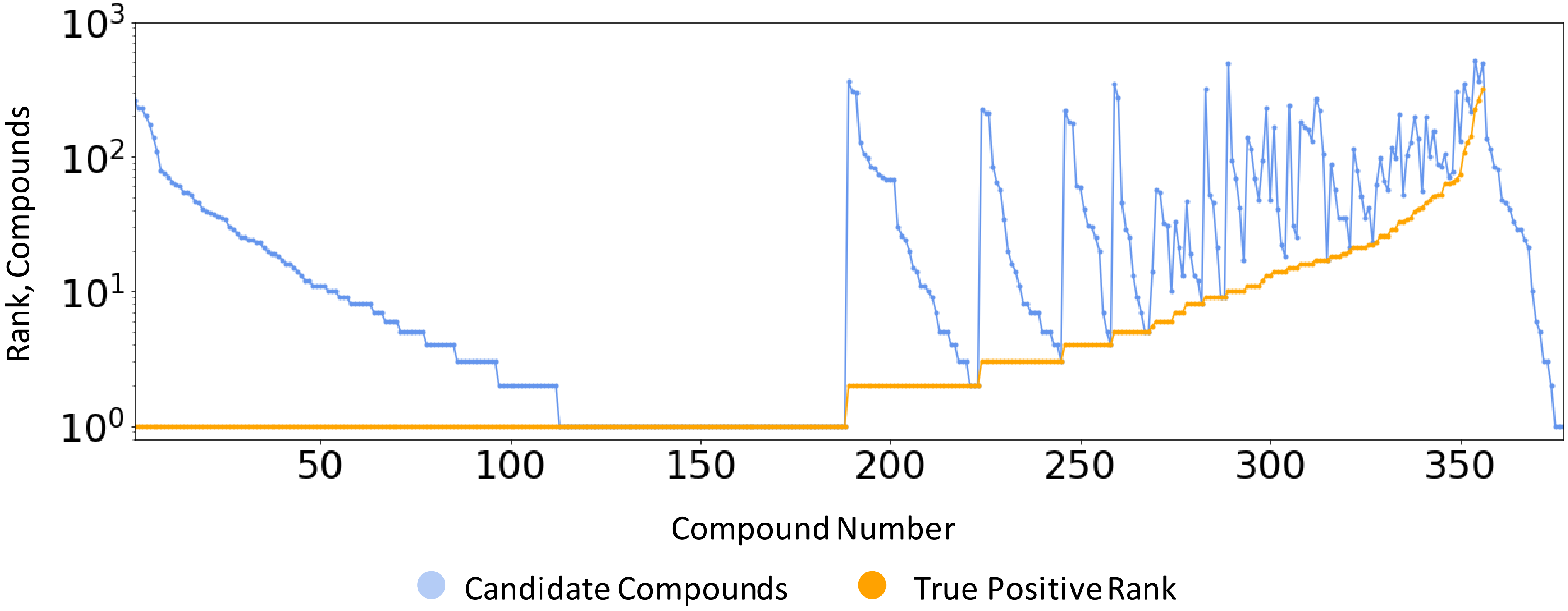

Figure S5B: Results of CFM-ID scoring for all “pass” compounds (n=377) when queried by mass and filtered by molecular formula. For each vertical pair, the blue point represents the number of retrieved candidate compounds for a given formula match, and the orange point represents the rank of the True Positive (TP) compound. Data are sorted by rank (increasing) and then number of candidate compounds (decreasing). “Pass” compounds without an associated scoring rank had insufficient fragment matches between the experimental and predicted spectra, and therefore, no match score

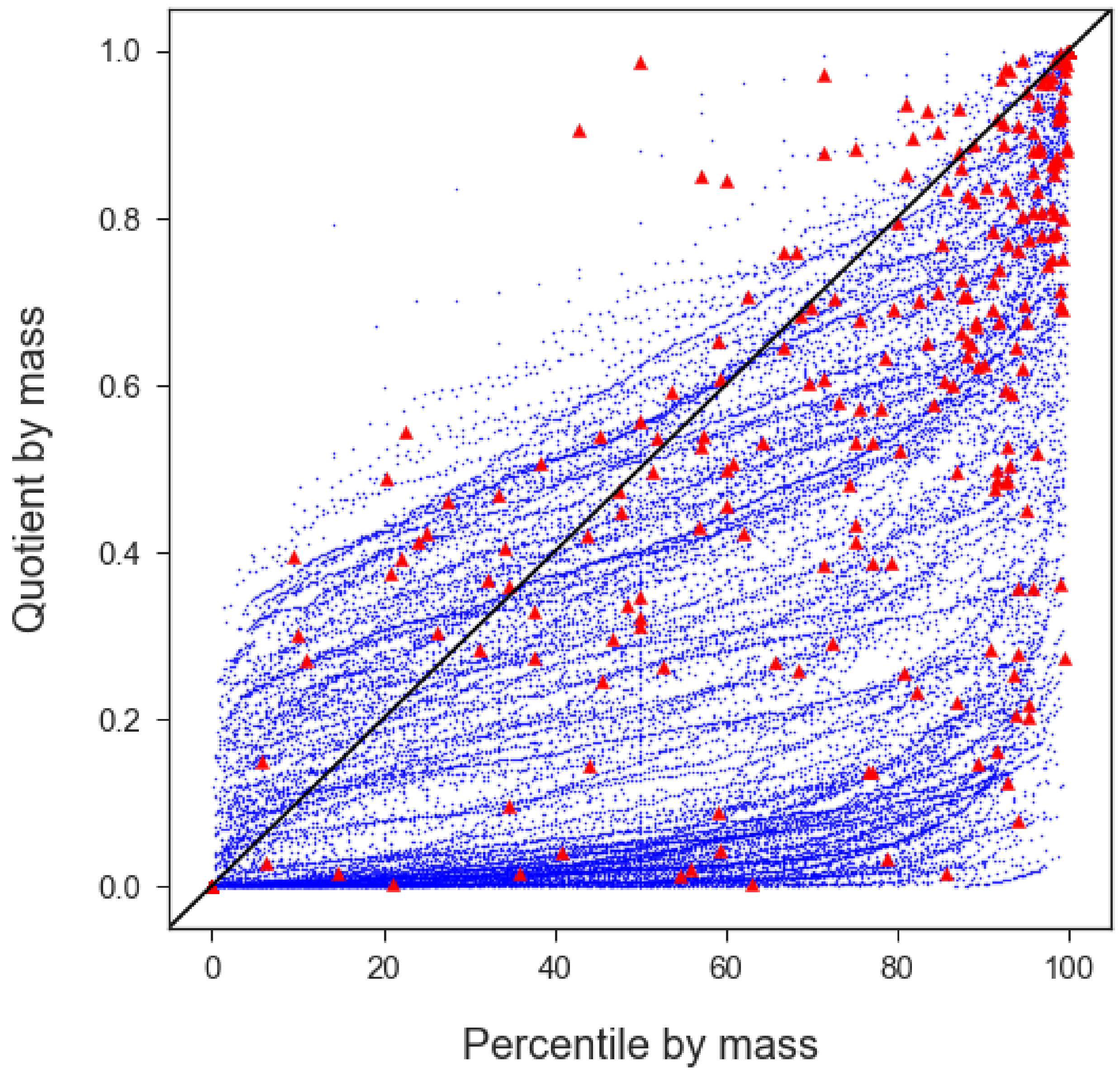

Figure S6. Scatterplot of quotient values vs. percentile values for all candidate compounds with a CFM-ID match score. The majority of values, for both “True Positive” (red triangles) and “Other Candidate” (blue circles) compounds, are below the diagonal line, indicating a less-than proportional increase in quotient values with rising percentile values. This trend reflects the uniform distribution of percentile values vs. right-skewed distribution of quotient values

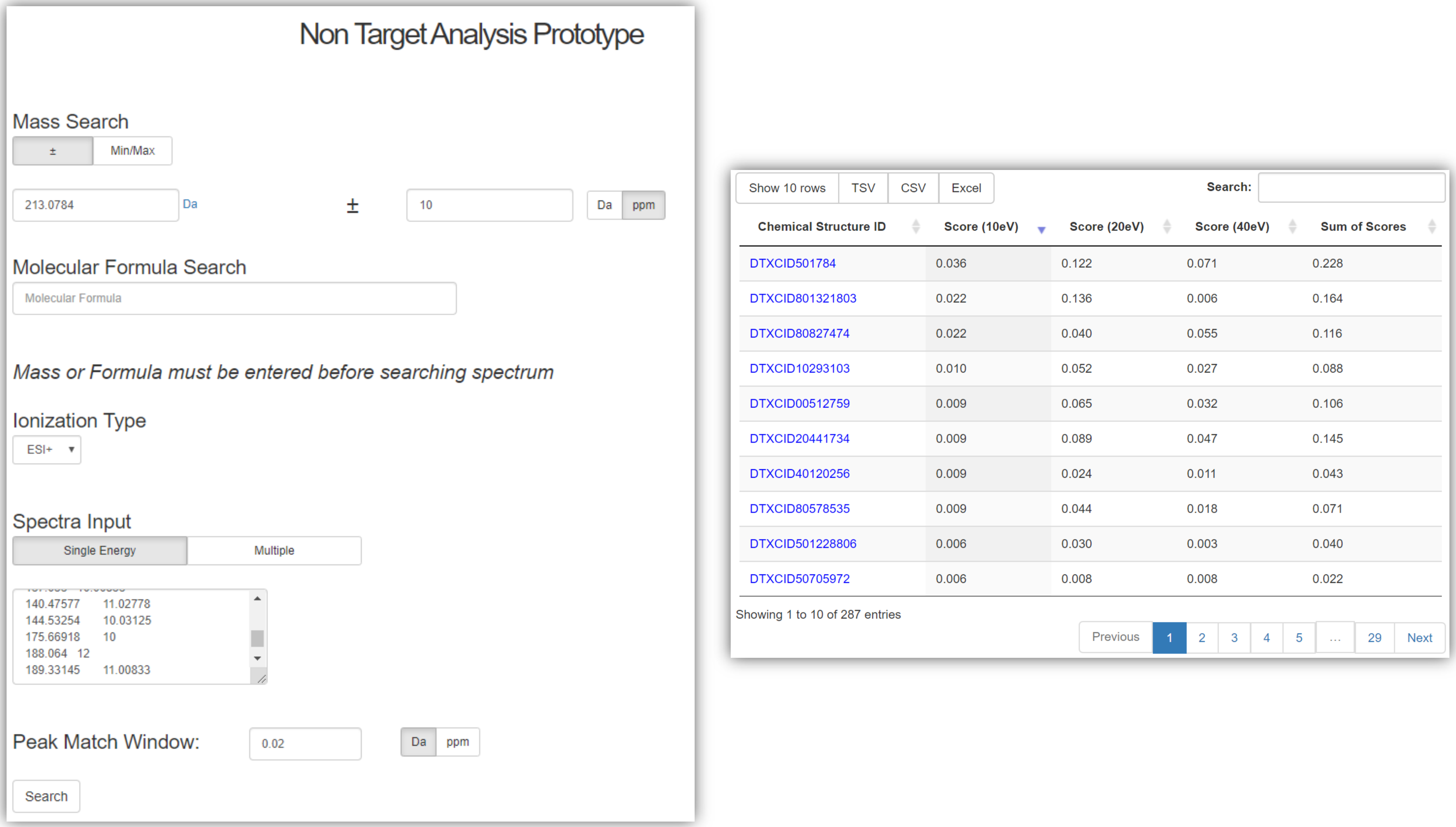

Figure S7A: Input page (left) and search results page (right) for a prototype web-based tool for searching experimental data against the CFM-ID database. The search requires: 1) an input neutral monoisotopic mass or molecular formula; and 2) experimental MS2 data in the format [fragment m/z, fragment intensity] for each line in the input field. One or multiple energies of experimental MS2 data may be entered. The result of the search is a table with all candidate compounds matched to the input mass or formula (identified by MS-Ready DTXCID) along with CFM-ID scores for each $CE_{in\ silico}$. Each candidate result is linked to a visualization page for the experimental and predicted spectra

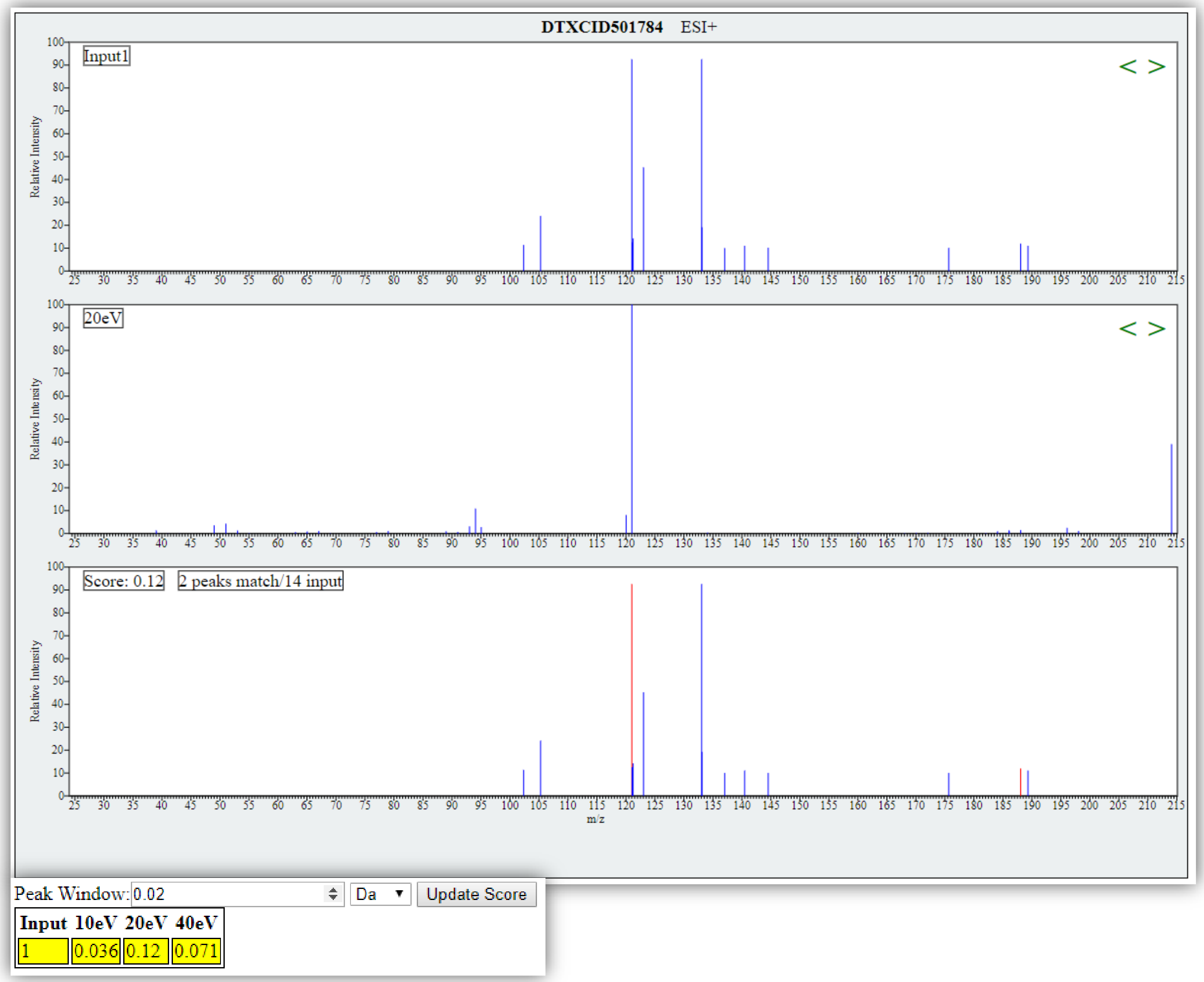

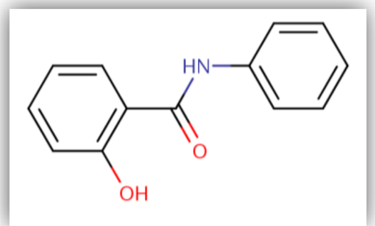

Salicylanilide

Figure S7B: Visualization page of experimental and predicted spectra for a candidate compound using the prototype web-based tool. Displayed on the page are the input experimental spectrum (top), predicted spectrum (middle), and spectrum comparison (bottom). The spectrum comparison is a copy of the input experimental spectrum with regards to spectral peaks and intensities; ions appearing as red are those which have been matched to an ion in the predicted spectrum. Each spectrum is scalable and scrollable through CE levels
